# Differential sparse attention framework for accurate ovarian cancer subtype classification using DenseNet121

**DOI:** 10.3389/fdgth.2026.1875306

**Published:** 2026-10-08

**Authors:** Muhammad Shoaib Saleem, Javed Rashid, Turke Althobaiti, Faiza Jameel, Khiati Zakaria, Hari Mohan Rai, Anwar Ghani

**Affiliations:** 1 Department of Mathematics, University of Okara, Okara, Punjab, Pakistan; 2 Center for Theoretical Physics, Khazar University, Baku, Azerbaijan; 3 Department of Computer Science, University of Okara, Okara, Pakistan; 4 Department of Computer Science, Faculty of Science, Northern Border University, Arar, Saudi Arabia; 5 Department of Computer Science and Information Technology, Abu Dhabi University, Abu Dhabi, United Arab Emirates; 6 School of Computing and Artificial Intelligence, Nazarbayev University, Astana, Kazakhstan

**Keywords:** cancer subtype, convolutional neural networks (CNN), deep learning, differential sparse attention, histopathology, medical image analysis, ovarian cancer

## Abstract

**Introduction:**

Ovarian cancer is the most fatal gynecologic cancer, contributing to approximately 207,000 deaths per year worldwide, largely due to late-stage diagnosis and considerable histopathological diversity across subtypes. Accurate subtype classification is clinically important for prognosis and treatment planning, but remains challenging due to high inter-class morphological similarity and the scarcity of adequately labeled histopathological datasets.

**Methods:**

This study presents a novel attention module, Deep Differential Sparse Attention (DDSA), designed for multi-class classification of ovarian cancer subtypes in histopathology images. The module selectively amplifies discriminative tumor regions while suppressing irrelevant background features, thereby improving both feature representation and model interpretability. A dataset of 3,082 images spanning five subtypes i.e., Clear Cell Carcinoma, Endometrioid Carcinoma, High-Grade Serous Carcinoma, Low-Grade Serous Carcinoma, and Mucinous Carcinoma--was obtained from Kaggle. The training set was balanced to 5,940 images (1,188 per subtype) using RandomOverSampler, with on-the-fly data augmentation applied during training. DDSA was integrated into a DenseNet-based architecture, and diagnostic performance was evaluated using standard classification metrics, including precision, recall, F1-score, confusion matrices, and ROC curves.

**Results:**

The proposed model achieved an overall classification accuracy of 92.53%, with a macro-average recall of 91.54% and a macro-average F1-score of 0.90. AUC values ranged from 0.92 to 0.99 across all five subtypes, indicating strong and consistent classification performance on the test set.

**Discussion:**

These results suggest that attention-guided DenseNet architectures can enhance discriminative feature extraction and improve diagnostic accuracy for ovarian cancer subtype classification. The DDSA module's ability to highlight tumor-relevant regions also supports improved model interpretability, an important consideration for clinical adoption. While these findings are promising, further validation on larger, multi-institutional datasets is needed to confirm robustness and generalizability, and to assess the framework's readiness for clinical deployment.

## 1 Introduction

Ovarian cancer remains one of the deadliest gynecologic cancers, with devastating effects on women’s health globally. The World Health Organization (WHO) reports that there are an estimated 314,000 new ovarian cancer diagnoses annually, with 207,000 reported deaths globally (1). Cancer is mainly diagnosed in its advanced stages since the initial symptoms are non-specific and ambiguous, leading to poor survival rates. It is estimated that as low as 20% of cases of ovarian cancer are diagnosed early, an element that has far-reaching effects on the treatment and survival rates (2). This underscores the need for advanced diagnostic methods to enable earlier and more accurate detection, which is crucial to improving patient survival. Furthermore, recent reports suggest that global mortality is expected to rise by 42% over the next decade if effective early-detection strategies are not implemented (3).

Epidemiologically, ovarian cancer shows considerable variations in incidence and survival rates across different areas. The highest incidence rates are observed in high-income countries, including the United States, Canada, and European countries, reaching up to 11.4 cases per 100,000 women per year. On the other hand, there are low rates in African and Asian countries, ranging from 4.5 to 7.2 per 100,000 women. The comparatively lower prevalence in these nations is reported, but middle- and low-income nations have lower survival rates, ranging from 30 to 40%, compared to over 50% in high-income countries. The survival differences underscore the importance of early detection, adequate facilities, and access to advanced diagnostic methods, which significantly affect treatment effectiveness (4). According to GLOBOCAN 2022, ovarian cancer mortality has declined in North America but continues to rise in South-Central Asia due to delayed diagnosis and lack of screening programs (5).

Ovarian cancer is histopathologically extremely diverse, with a series of discrete types: Clear Cell Carcinoma (CC), Endometrioid Carcinoma (EC), High-Grade Serous Carcinoma (HGSC), Low-Grade Serous Carcinoma (LGSC), and Mucinous Carcinoma (MC). Molecular and clinical characteristics of each type determine treatment and prognosis. Such subtypes must, in turn, be appropriately categorized as subtypes so that they can plan a particular treatment course and anticipate the outcome for each patient (6). Diagnostic results are inconsistent because histological analysis is highly dependent on pathology and on observer variability in standard classification. Such problems highlight the need for more efficient and fair methods of ovarian tumor classification. Artificial intelligence in digital pathology has already significantly enhanced the detection of variations across ovarian cancer subtypes, according to recent studies (7). It has recently been proposed that combining machine learning and genomics may improve prognostic value and diagnostic quality (8).

Despite advances that have been made in the study of ovarian cancer, gaps remain in early detection and accurate classification of subtypes, posing significant challenges. The conventional histopathological methodology is subjective, time-consuming, and observer-biased; hence, it is of limited value. Moreover, morphological overlap of subtypes of ovarian cancer complicates classification in the sense that it is hard to identify the two subtypes accurately (9). Besides this, conventional diagnostic methods do not necessarily lead to reproducible outcomes, particularly in rare or unusual cases (10).

Artificial intelligence (AI) and deep learning (DL) are the solutions to these problems. Deep learning models, particularly CNNs, have made tremendous progress in medical imaging, including cancer diagnosis (11). Recently, Transformer-based models and attention mechanisms have been applied to histopathology images, outperforming classical CNNs in feature extraction and generalization (12). Additionally, federated learning approaches have been introduced to enable collaborative model training across institutions while preserving patient privacy (13).

Deep learning models can automatically identify delicate patterns in histopathological images from large datasets that may be beyond the ability of human pathologists. However, despite their successes, deep learning models for ovarian cancer diagnosis still face challenges such as class imbalance, low generalizability across datasets, and a lack of interpretability. Class imbalance occurs when some subtypes are underrepresented in the training dataset, leading to biased model predictions that are less accurate for rarer subtypes (14). Moreover, the complex “black-box” nature of deep learning models is a barrier to their clinical adoption, as healthcare professionals require transparent models that provide understandable reasons for their decisions (15).

Recent explainable AI (XAI) methods, such as Grad-CAM++ and Integrated Gradients, have shown promise but have not yet been used for the diagnosis of ovarian cancer (16). This work addresses the limitations outlined above by proposing a deep learning approach. A pretrained CNN-based model with a Differentiated Attention mechanism. The proposed model aims to enhance the performance of classification based on the consideration of selective features in histopathology images and enhance the interpretability of the model to assist in clinical decision-making. Additionally, oversampling and data augmentation are employed to ensure that each ovarian cancer subtype receives the same level of training and to minimize inter-class distance (17). In addition, the research should be based on AI models, clinical data, and imaging biomarkers to enhance practical generalizability (18). To generate the most accurate categories, this study demonstrates that deep learning can be applied further to subdivide ovarian cancer.

A pretrained CNN and Differential Attention are employed to apply data augmentation and oversampling to mitigate class imbalance and to retain significant features. In addition, the model’s attention mechanisms are easier to understand and facilitate its use in clinical practice. Finally, the article illustrates that precision oncology with AI has the potential to improve the diagnosis and treatment of ovarian cancer (19). The five most relevant subtypes used in this study were histological images of ovarian cancer. Oversampling and data augmentation help minimize class imbalance, and the model is evaluated using multiple performance metrics. The model’s generalizability and its application in real-world settings will be demonstrated using external data and clinical data in the following stages of the research (20).

The summary of the work is as follows: the most important contribution of the research:

- Introduces a multi-class DenseNet121-based deep learning framework to classify ovarian cancer subtypes using histopathological images using a Differentiated Sparse Attention (DSA).
- A special attention layer is designed to enhance the discrimination of the discriminative tumor areas and suppress irrelevant background features to enhance classification accuracy and robustness.
- A manually balanced dataset was constructed to overcome the issue of imbalance in the classes and ensure reliable learning in all five subtypes of ovarian cancer (CC, EC, HGSC, LGSC, and MC) through appropriate augmentation and oversampling.

The following paper is organized as follows. The literature review is in Section 2, the data set and pre-processing are in Section 3, results and discussion are in Section 4, and lastly, a conclusion is in Section 5 where future directions are discussed.

## 2 Literature review

Ovarian cancer has been classified as one of the deadliest gynecological cancers because of its late-stage diagnosis and its histopathological complexity.While the treatment strategies are now better, the five-year survival rate is still low, indicating the need for better diagnostic methods, timeliness of diagnosis and individual treatment plans. Although the accuracy of early-stage ovarian cancer detection using AI-assisted diagnostic models has already reached 80%–86%, there is still a need to further improve predictive modeling (21).

In recent years, AI, in particular DL, has been prominently involved in the diagnosis of ovarian cancer. In particular, CNNs have been shown to be very successful in the classification of histopathological images among these methods. Developed a multi-class classification model of the ovarian cancer histotypes using a CNN with an approximate accuracy of 85%. Their approach adequately distinguishes ovarian cancer histotypes but lacks modeling of global context, which is central to understanding complex tissue variations (22). To address the interpretability gap in deep learning models, researchers have been using Explainable AI (XAI) methods.

Using XAI, a machine learning system provides a graphical explanation of the model’s predictions. Transparency, computerized diagnosis, and clinical confidence in automated decision-making are among its benefits (23). The classification of ovarian cancer is made even more difficult by the asymmetrical form of the classifications that usually occur in medical data. The highest-quality data were found to be crucial for the application of AI in diagnostics. To overcome this limitation, (24) improved preprocessing and feature selection techniques, achieving high-quality results and increasing test accuracy from 73.53% to 83.03% and from 92% to 92%.

AI has expanded beyond histopathology to include applications in liquid biopsy. An artificial intelligence (AI) system to forecast the course of ovarian cancer using blood biomarkers and circulating tumor cells (CTCs). Ma et al. (25), with excellent performance in clinical staging and treatment-sensitivity prediction, this non-invasive method produced encouraging results for real-time monitoring. At the same time, AI techniques are already contributing to personalized therapy planning. The use of AI for the prediction of chemoresistance as well as the direction of individualized treatment protocols. Their study reveals that conventional therapy regimens are being replaced by precision oncology, in which therapy is tailored to imaging and molecular signatures (26). At the same time, other machine learning tools such as XGBoost have also proven to be very good at detecting early-stage ovarian cancer with up to 80% on formatted clinical data (27).

CNNs on multi-class histopathology images performed equally well on deep networks, showing that complex tissue morphology could be recognized with CNNs (10). In the same case, (25) combined explainable deep learning methods, including Grad-CAM, to enhance interpretability in multi-class ovarian cancer prediction. InceptionV3 classifiers have also achieved high subtype classification accuracy by employing transfer learning to improve generalization across histological variations (28). Treatment response prediction has also been investigated using structured patient records. The results reported by (29) (logistic regression models trained on over 1,000 HGSC patients) demonstrate the feasibility of simple, interpretable models for clinical decision-making, with an AUC of 74.1%. Similarly, AI models that analyzed CTC features confirmed the value of liquid biopsy for personalized care, with AUCs of 79.6% for clinical stage prediction and 80.9% for platinum sensitivity (25).

Methods such as Random Forest (RF) and Support Vector Machines (SVM) have been used in biomarker discovery to analyze gene expression datasets and identify predictors of response to platinum-paclitaxel therapy. These results validate personalized treatment based on biomarkers (9). To classify histopathological images into subtypes EC, CC, MC, HGSC, and LGSC, supervised learning algorithms have been applied, achieving up to 75% accuracy, which validates the diagnostic usefulness of machine learning for subtype differentiation (27).

Reliability was further enhanced by ensemble models (30) that stacked ResNet-50 on a Kaggle dataset in the OCEAN AI contest, achieving a maximum accuracy of 89%, demonstrating the usefulness of hybrid methods for classifying ovarian cancer. Nevertheless, even with these developments, the majority of existing models still have problems with the classification of rare subtypes, over-reliance on a single modality, and limited generalizability, underscoring the need to establish clinically interpretable, multi-modal, and deployable AI systems. Despite the advancements in the field of AI-powered ovarian cancer diagnosis, there is still significant room for improvement. The majority of the existing models have an accuracy of 75%–85%, but they are not effective at classification of rare subtypes due to class imbalance. The majority of studies solely rely on one modality of data and do not take advantage of multimodal fusion of histopathology, biomarkers and clinical data. Although XAI techniques are available, there are no clinically interpretable and deployable solutions. Most models are also trained with a small or niche dataset, and the generalizability and real-world applicability is questionable. AI systems that are also efficient and interpretable, and are user friendly in real time clinical settings, particularly in resource-limited areas, are also essential.

Table 1 illustrates not only the accuracy of each AI technique, but also shows an increasing trend of incorporating model interpretability and data processing methods. Previous work was focused on the accuracy of the results, but recent work, for example using XAI frameworks or recursive feature elimination, was focused on performance and transparency. Furthermore, the comparison reveals that ensemble and attention based models are more accurate in multi-class problem and especially on real-world datasets like Kaggle. These advancements represent a new paradigm in research that is not only identifying the issues, but now more advanced, explainable, and clinically adaptable solutions for ovarian cancer diagnosis.

**Table 1:** Comparison of AI and machine learning methods for ovarian cancer classification and prediction.

Reference	Method	Dataset	Classes	Accuracy	Pros and cons
(21)	AI-assisted Diagnosis	Clinical Data	Early-stage Ovarian Cancer Detection	80%–86%	Focuses on early detection, No subtype analysis included
(22)	CNN (Deep Learning)	Histopathological Images	Multi-class Ovarian Cancer Subtypes	85%	High accuracy for tissue features, Limited context modeling
(23)	ML + Preprocessing + RFE	Histopathology Images	Subtype Classification (EC, CC, MC, HGSC, LGSC)	73.53–83.03% (Test), 92% (RFE)	Handles class imbalance well, Heavy preprocessing required
(26)	AI for Chemoresistance Prediction	Structured Clinical Data	Therapy Planning (Chemoresistance)	No specific accuracy reported	Personalized therapy planning, No model accuracy shared
(27)	XGBoost	Structured Clinical Data	Early-stage Ovarian Cancer Detection	80%	Interpretable and simple, No imaging data used
(10)	DL (CNNs)	Multi-class Histopathology Dataset	Ovarian Cancer Subtypes	Similar to 80%	Auto feature extraction, Needs large datasets
(28)	Logistic Regression	1,002 HGSC Patient Records	Platinum Sensitivity Prediction	74.1% (AUC)	Simple and interpretable,Less powerful than deep models
(29)	AI on CTC Biomarkers	CTC Data	Clinical Stage & Platinum Sensitivity	79.6% (Stage ROC), 80.9% (Platinum ROC)	Confirms value of liquid biopsy, Biomarker limitation
(25)	RF + SVM	Gene Expression (GEO Dataset)	Platinum-Paclitaxel Therapy Prediction	Not specified	Useful for biomarker discovery, Accuracy not mentioned
(30)	ResNet50 + SVM + Stacking Ensemble	Kaggle Dataset (349 Patients)	Ovarian Cancer Positive/Negative & Multi-class Subtype Classification	75% (ResNet50), 85% (SVM), 89% (Ensemble)	High accuracy via ensemble, Computationally intensive

Although the present study is restricted to the classification of subtype by histopathology, it is worthwhile to place the proposed framework in the context of the broader field of AI-powered ovarian cancer imaging, with recent developments in the use of ultrasound for diagnosis. These modalities are not direct comparators to histopathology models; however, they offer valuable methodological lessons regarding patient-level validation, external generalisability, uncertainty quantification, and clinical deployment that are directly relevant to the limitations and future directions of the present work.

Recent ultrasound-based AI studies have demonstrated that rigorous multi-centre validation is both feasible and necessary for clinical adoption. The researchers (31) conducted an international multi-centre validation of an AI-driven ultrasound system for ovarian cancer detection across diverse patient populations and imaging equipment, reporting robust performance that generalised across institutions—a standard of external validation that histopathology-based models, including the present work, have yet to achieve. Similarly, the authors (32) evaluated a deep learning ultrasound assistant in a multi-centre clinical setting, demonstrating that AI-assisted diagnosis improved clinician performance when the model was embedded within a real workflow and subjected to patient-level rather than image-level evaluation. These studies underscore that single-dataset, patch-level accuracy metrics—as reported in most histopathology classification studies including the present one—are insufficient to establish clinical utility, and that prospective multi-institutional testing at the patient level is an essential next step.

Systematic reviews of the ultrasound-AI literature further highlight persistent challenges that are shared across imaging modalities. Garcia-Atutxa et al. (33) conducted a comprehensive systematic review and quantitative assessment of AI models for ultrasound-based ovarian cancer diagnosis, identifying model calibration, explainability, and dataset heterogeneity as the three most critical barriers to clinical translation. These findings are directly applicable to histopathology-based deep learning models: the present model’s softmax outputs have not been calibrated, and formal explainability—in the form of pathologist-reviewed attention maps or Grad-CAM visualisations—remains a future objective rather than a current achievement. Li et al. (34) performed a meta-analysis of AI ultrasound systems for initial malignancy diagnosis, reporting pooled sensitivities and specificities that exceeded those of radiologists alone, but also noted high heterogeneity across studies attributable to differences in dataset size, class balance, and evaluation methodology—issues equally relevant to the histopathological classification literature.

At the architectural level, Garcia-Atutxa et al. (35) proposed early-fusion hybrid CNN-Transformer models for multiclass ovarian tumour classification from ultrasound, demonstrating that combining local convolutional feature extraction with global self-attention improves discrimination among morphologically overlapping tumour types. This architectural insight is consistent with the motivation of the present DSA module, which similarly integrates spatial attention into a convolutional backbone to address inter-class morphological similarity in histopathological subtypes. Collectively, these ultrasound-AI studies suggest that attention-based and transformer-augmented architectures represent a convergent methodological direction across ovarian cancer imaging modalities, and that the primary remaining challenge is not architectural innovation but rigorous clinical validation—including external datasets, patient-level aggregation, uncertainty quantification, and expert pathological review of model decisions.

## 3 Material and methods

Timely diagnosis of the ovarian cancer subtypes is essential to ensure timely treatment and better outcomes. A new DL architecture, DenseNet-121 with DSA, is proposed to improve feature learning and reduce background noise. The general scheme of the suggested methodology is depicted in Figure 1.

**Figure 1:**
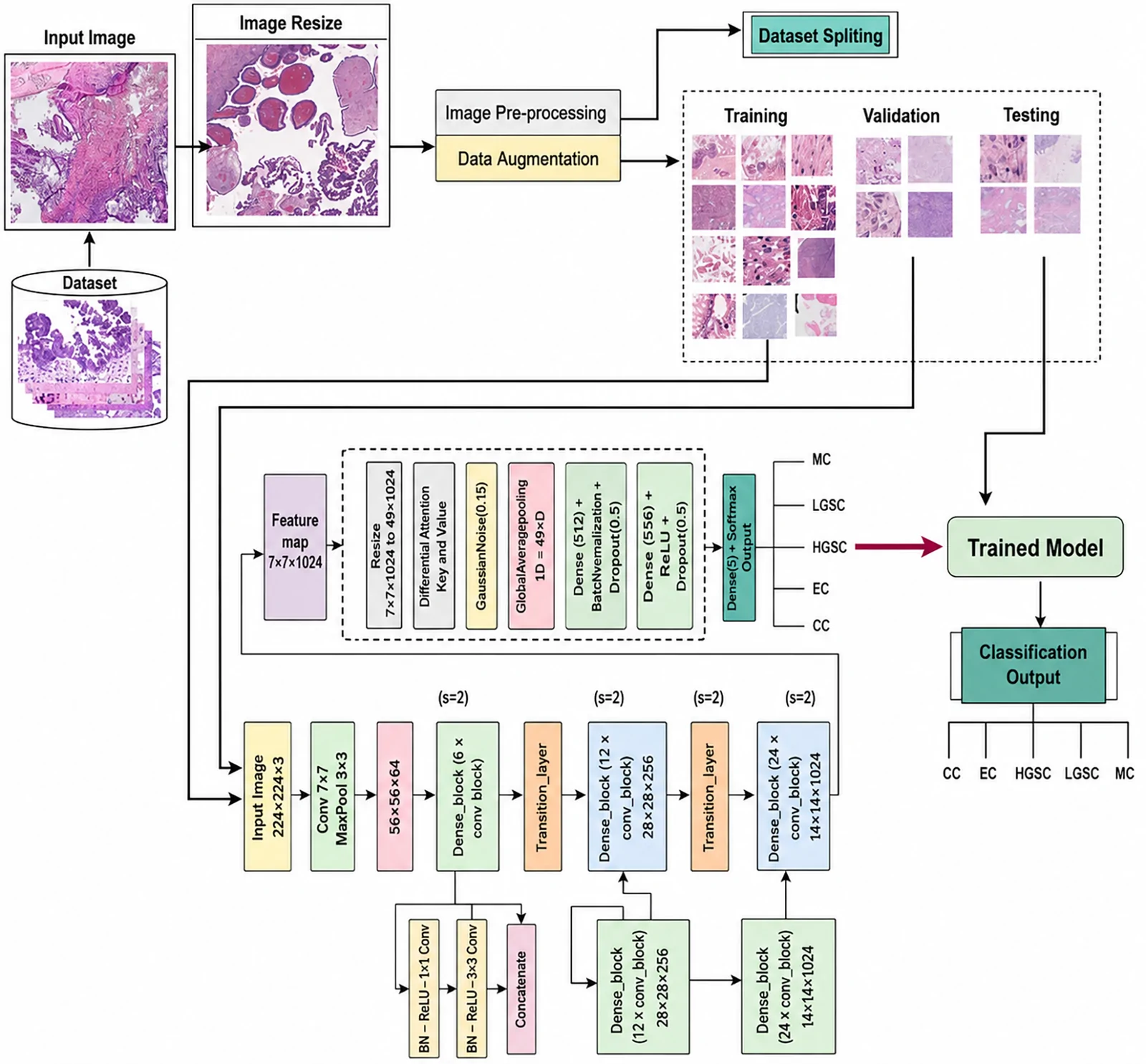
The flow diagram of the proposed method.

### 3.1 Dataset

In this work, an array of epithelial ovarian cancer types was categorized based on a set of histopathological 2D images. The data (3082 images, five subtypes, including CC, EC, HGSC, LGSC, and MC) were obtained in a publicly accessible Kaggle (36). These images were derived from digital histopathology slides and captured the morphological differences among cancer subtypes. To correct this imbalance, the underrepresented classes (minorities) were artificially overrepresented by the *RandomOverSampler* method. This resulted in a homogeneous dataset of 5,940 pictures, 1,188 images per category. Ensuring equal representation across subtypes and enhancing the classification model’s generalizability required this preprocessing step. The distribution of classes in the original dataset was highly imbalanced, as shown in Table 2, with HGSC accounting for most samples, whereas LGSC and MC were grossly underrepresented. The source dataset does not provide patient, specimen, or slide-level identifiers; images are distributed as independently labeled files organized by subtype, without accompanying case-level metadata. Consequently, the image-based split performed in this study cannot guarantee patient-level separation between training, validation, and test sets. This constraint is inherent to the publicly available dataset and is consistent with the image-level evaluation protocol adopted by prior studies using this same source [e.g., (37)]. This limitation is explicitly acknowledged in Section 5 (Conclusion and Future Work), where patient-level validation on an independent, identifier-rich cohort is proposed as a necessary next step.

**Table 2:** Description of the dataset without applying data augmentation.

Class labels	Images
HGSC	1,188
EC	733
CC	551
MC	323
LGSC	287
Total samples	**3,082**

Bold values represent the proposed approach.

### 3.2 Data preprocessing

The histopathological images were initially processed into a DataFrame with their respective ovarian cancer subtype labels (CC, EC, HGSC, LGSC, MC) using a structured preprocessing pipeline. Then, the class labels were numerically encoded using scikit-learn’s LabelEncoder to be used in the deep learning model. To avoid any problems with input dimensions differing across the data set, all images were resized to 224×224 images with 3 RGB channels. This resizing maintains the morphological and structural properties relevant to the classification of the subtypes and optimizes the use of memory when training.

The original 3,082-image dataset was first split (80/10/10%) by stratified sampling. RandomOverSampler was then applied exclusively to the 2,465 training images to balance minority classes, yielding 5,940 training images (1,188 per class). Validation and test sets were kept at their original unaugmented distributions.

#### 3.2.1 Data augmentation

To improve generalization and reduce overfitting, data augmentation was applied exclusively to the training set, whereas the validation and test sets were preprocessed only. We employed the Keras ImageDataGenerator with DenseNet’s preprocess_input normalization, which scales pixel values into the range of [−1,1] compatible with ImageNet pretrained weights. The augmentation pipeline comprised rotations of up to $\pm 20^{\circ }$, horizontal and vertical shifts of 0.1, zooming up to 0.2, and a mild shear value of 0.07. Variability in staining across different H&E slides was compensated for by adjusting brightness to 0.85–1.15. Horizontal and vertical flipping further improved invariance to orientation changes. Where empty pixels were created due to transformations, the reflect fill mode was used. These extensions had a beneficial effect, increasing the training range, simulating histopathological variation, and improving the generalizability of the proposed model to unseen test data. Table 3 provides a summary of the augmentation transformations and their corresponding settings. The operations improved the model’s generalization to unseen test data and mitigated class imbalance by synthetically diversifying underrepresented subtypes.

**Table 3:** Applied data augmentation techniques.

Transformation	Setting	Purpose
Preprocessing (DenseNet)	preprocess_input	Normalizes pixel values to [−1, 1], consistent with ImageNet pretrained weights.
Rotation	$\pm 20^{\circ }$	Improves rotational robustness by simulating orientation variability.
Zoom	0.20	Enhances pattern recognition by emulating magnification changes.
Width shift	0.10	Simulates horizontal displacement of tissue regions.
Height shift	0.10	Simulates vertical displacement in the image field.
Shear	0.07	Introduces mild affine distortion to reflect morphological variability.
Brightness adjustment	0.85–1.15	Accounts for variations in H&E staining intensity across slides.
Horizontal & vertical flip	True	Increases variation and reduces overfitting by changing image orientation.
Fill mode	Reflect	Fills missing pixels introduced by geometric transformations.

#### 3.2.2 Data splitting

The data used in this paper comprised 3,082 histopathological images of five types of ovarian cancer: CC, EC, HGSC, LGSC, and MC. Stratified splitting was performed first on the original dataset. Data augmentation was applied only to the training set post-split. Validation and test sets received only DenseNet preprocessing normalization.The dataset was split into three subsets: training (80%), validation (10%), and testing (10%).

The dataset consists of 5,940 images for training after oversampling (with on-the-fly augmentation applied during training), along with 308 images for validation and 308 images for testing. This split was planned to provide strong model training and unbiased performance evaluation. Stratification was used to avoid class imbalance, especially the predominance of highly represented classes like HGSC, to make sure each of the classes were fairly represented in each subtype. This approach promotes stable learning and enhances the generalization capability of deep learning models. The detailed sample distribution is presented in Table 4 and Figure 2.

**Table 4:** Class-wise sample distribution after stratified splitting and training-set oversampling.

Subset	CC	EC	HGSC	LGSC	MC	Total
Training	1,188	1,188	1,188	1,188	1,188	5,940
Validation	55	73	119	29	32	308
Testing	55	73	119	29	32	308

Splitting was performed first on the original 3,082-image dataset; RandomOverSampler was then applied exclusively to the training subset to reach 1,188 images per class. On-the-fly data augmentation (Table 3) was subsequently applied only during training and does not alter the reported counts; validation and test sets received DenseNet preprocessing only and remain unaugmented.

**Figure 2:**
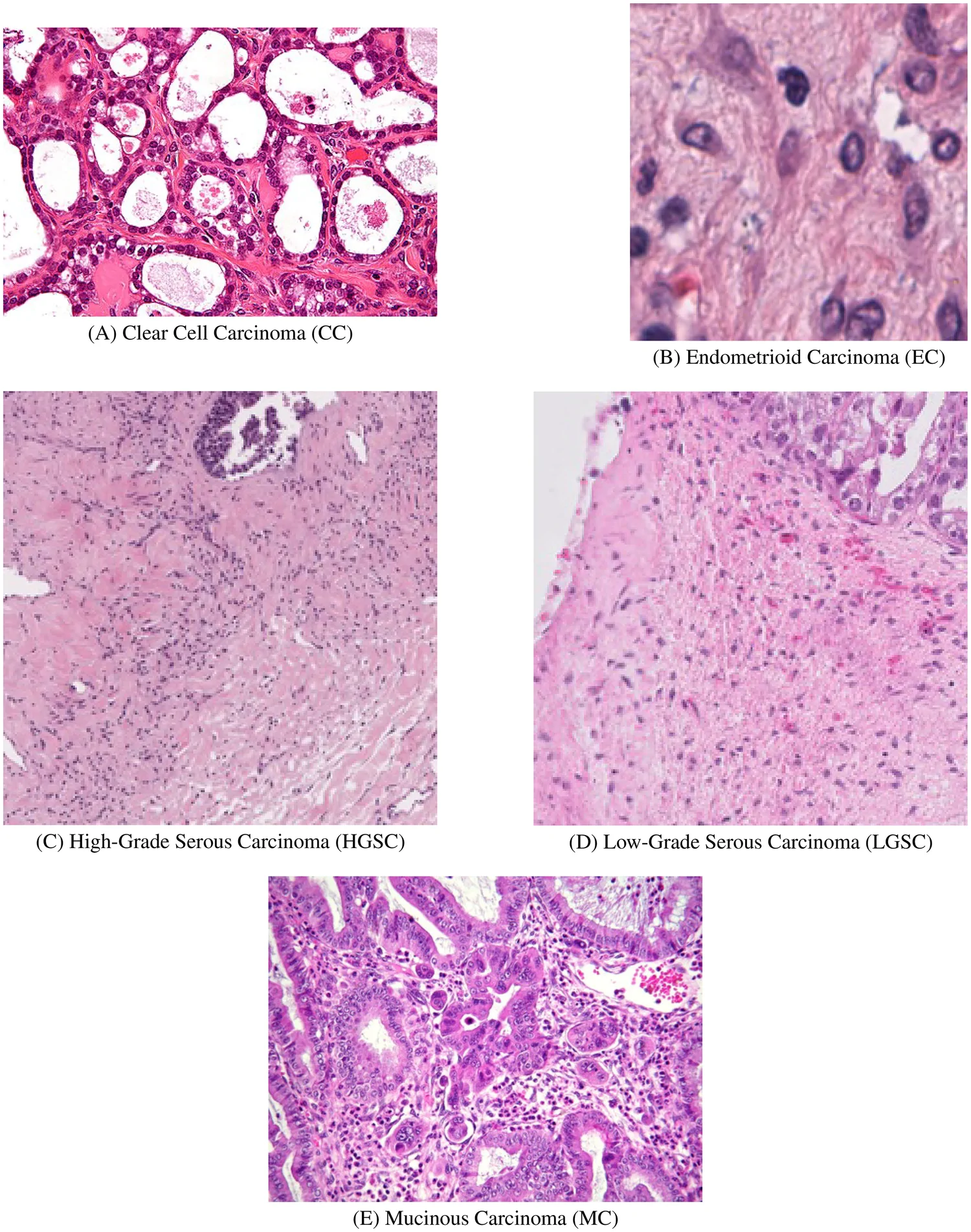
Histopathological images of ovarian cancer subtypes. **(A)** Clear Cell Carcinoma (CC). **(B)** Endometrioid Carcinoma (EC). **(C)** High-Grade Serous Carcinoma (HGSC). **(D)** Low-Grade Serous Carcinoma (LGSC). **(E)** Mucinous Carcinoma (MC). Images from “Ovarian Cancer Subtype Classification” dataset by Sunil Thite, licensed under Apache 2.0.

### 3.3 The proposed methodology

The proposed framework uses a DenseNet121 backbone with a DSA mechanism to classify histopathology images of ovarian cancer. The input image of dimension 224×224×3 is then fed through an initial 7×7 convolution and 3×3 max pooling to produce feature maps 56×56×64. The architecture comprises three sequentially deeper, dense blocks linked by transition layers. The initial dense block contains 6 convolutional layers, followed by a transition layer that reduces the spatial dimensions to 28×28×256. The second thick block is made up of 12 convolutional layers, and another transition layer downsamples the feature map to 14×14×512. The third dense block, composed of 24 convolutional layers, is preceded by a transition layer that produces 7×7×1,024 output feature maps.

#### 3.3.1 Differential sparse attention (DSA) module

At this level, the feature map (7×7×1024) is reshaped to $F\in R^{N\times d}$, where N=49 spatial positions and d=1024 channel dimensions. This reshaped tensor is then processed by the DSA mechanism.

##### 3.3.1.1 Query, key, and value projections

Three learned linear projections map the feature representation to a compact key dimension, Equation 1 $d_{k}=64$:

$$Q=FW_{Q},\;K=FW_{K},\;V=FW_{V}$$ (1)

where $W_{Q},\;W_{K},\;W_{V}\in R^{d\times d_{k}}$ are trainable projection matrices.

##### 3.3.1.2 Differential attention computation

The core innovation of the DSA module is the computation of two attention distributions at different temperature scales, Equation 2 and Equation 3 $\tau_{1}=1.0$ and $\tau_{2}=0.5$:

$$A_{1}=\text{softmax}\left(\frac{QK^{⊤}}{\sqrt{d_{k}}\;\tau_{1}}\right),\;A_{2}=\text{softmax}\left(\frac{QK^{⊤}}{\sqrt{d_{k}}\;\tau_{2}}\right)$$ (2)

$$A_{\text{diff}}=A_{1}-A_{2}$$ (3)

Note that dividing the logits by τ (rather than multiplying) follows the standard temperature-scaling convention: since $\tau_{2}=0.5<\tau_{1}=1.0$, dividing by the smaller $\tau_{2}$ amplifies the logit magnitude, producing a sharper, more peaked distribution $A_{2}$ concentrated on dominant positions, while $A_{1}$ remains a more diffuse, general view of the attended region. Their element-wise subtraction $A_{\text{diff}}$ amplifies positions where the model holds strong discriminative confidence while cancelling diffuse, uninformative background attention. Because both $A_{1}$ and $A_{2}$ are row-stochastic (each row sums to 1), every row of $A_{\text{diff}}$ sums to exactly zero, meaning it necessarily contains both positive and negative entries by construction.

##### 3.3.1.3 Sparsity enforcement

To further suppress irrelevant spatial regions, a hard sparsity threshold θ=0.01 is applied element-wise in Equation 4 to $A_{\mathrm{diff}}$:

$${\tilde{A}}_{\mathrm{diff}}^{\left(i,j\right)}=\left\{ \begin{array}{cc} A_{\mathrm{diff}}^{\left(i,j\right)}\; & \text{if }A_{\mathrm{diff}}^{\left(i,j\right)}>\theta \;\\ 0\; & \text{otherwise}\; \end{array}\right.$$ (4)

Only spatial positions where the sharper distribution $A_{2}$ concentrates more strongly than the diffuse distribution $A_{1}$ are retained; thresholding at θ>0 discards both negative entries (introduced by construction, since rows of $A_{\text{diff}}$ sum to zero) and small positive entries, yielding a sparse map that directs the model’s attention toward diagnostically relevant tumor regions. Because row-sums are no longer preserved after thresholding, ${\tilde{A}}_{\text{diff}}$ should be interpreted as a learned relevance weighting rather than a probability distribution.

##### 3.3.1.4 Output with residual connection

The projected value tensor $V\in R^{N\times d_{k}}$ (with $d_{k}=64$) has a lower channel dimension than the original feature tensor $F\in R^{N\times d}$ (with d=1024), so a direct residual addition is dimensionally invalid. To resolve this, an output projection $W_{O}\in R^{d_{k}\times d}$ maps the attended values back to the original channel dimension before the residual connection is applied (Equation 5):

$$O=\left({\tilde{A}}_{\text{diff}}\;V\right)W_{O}+F$$ (5)

where $O\in R^{N\times d}$, matching the shape of F. The residual connection preserves the original feature information and stabilises gradient flow during fine-tuning, while $W_{O}$ ensures the attended representation is correctly aligned with the backbone’s channel dimensionality before being added back.

##### 3.3.1.5 Trainable parameters

The DSA module adds three input projection matrices $W_{Q},W_{K},W_{V}\in R^{1024\times 64}$, each contributing 1024×64+64=65,600 parameters (including bias terms), plus one output projection matrix $W_{O}\in R^{64\times 1024}$ required to restore the residual-compatible channel dimension, contributing 64×1,024+1,024=66,560 parameters. The total added parameters are (Equation 6):

3×(1024×64+64)+(64×1,024+1024)=196,800+66,560=263,360 parameters (6)

Relative to the DenseNet121 backbone (≈7.04 million parameters), this represents approximately 3.74% overhead, confirming that DSA remains a lightweight addition while delivering measurable classification gains, as demonstrated in the ablation study (Section 4.2.1, Table 5).

**Table 5:** Controlled ablation study showing the contribution of each component.

Configuration	Accuracy	Macro F1	LGSC Recall	HGSC Recall
DenseNet121 (Baseline)	84.20 ± 0.42	0.831 ± 0.006	0.724 ± 0.011	0.903 ± 0.007
DenseNet121 + Standard Attention	87.60 ± 0.35	0.869 ± 0.005	0.759 ± 0.010	0.924 ± 0.006
DenseNet121 + Differential Attention (without sparsity)	90.90 ± 0.27	0.892 ± 0.004	0.854 ± 0.008	0.937 ± 0.005
DenseNet121 + Differential Sparse Attention (DSA)	92.53 ± 0.18	0.900 ± 0.003	0.878 ± 0.007	0.942 ± 0.004
DenseNet121 + DSA (without Gaussian Noise)	89.30 ± 0.30	0.881 ± 0.004	0.841 ± 0.009	0.929 ± 0.005
DenseNet121 + DSA (without Oversampling)	86.80 ± 0.39	0.847 ± 0.006	0.712 ± 0.013	0.933 ± 0.006
DenseNet121 + DSA (without Data Augmentation)	90.60 ± 0.26	0.889 ± 0.004	0.848 ± 0.008	0.936 ± 0.005

Results are reported as Mean ± Standard Deviation over five independent random seeds.

Algorithm 1 **Differential Sparse Attention (DSA) Module.**

**Require:** Feature map $F\in R^{N\times d}$, N=49, d=1024

**Require:** Weights $W_{Q},W_{K},W_{V}\in R^{d\times d_{k}}$, $W_{O}\in R^{d_{k}\times d}$, $d_{k}=64$

**Require:** Temperatures $\tau_{1}=1.0$, $\tau_{2}=0.5$; threshold θ=0.01

 1: $Q\leftarrow FW_{Q}$; $K\leftarrow FW_{K}$; $V\leftarrow FW_{V}$

 2: $L\leftarrow \frac{QK^{⊤}}{\sqrt{d_{k}}}$               ▹ scaled logits, shape (N,N)

 3: $A_{1}\leftarrow \mathrm{softmax}(L/\tau_{1})$              ▹ diffuse distribution

 4: $A_{2}\leftarrow \mathrm{softmax}(L/\tau_{2})$              ▹ sharp distribution

 5: $A_{\mathrm{diff}}\leftarrow A_{1}-A_{2}$

 6: ${\tilde{A}}_{\mathrm{diff}}\leftarrow A_{\mathrm{diff}}\cdot \leftarrow [A_{\mathrm{diff}}>\theta ]$                 ▹ sparsify

 7: $O\leftarrow ({\tilde{A}}_{\mathrm{diff}}V)W_{O}+F$            ▹ project back & residual add

 8: **return** $O\in R^{N\times d}$

#### 3.3.2 Classification head

To further increase robustness, Gaussian noise (σ=0.15) is added to the DSA output O, and 1D Global Average Pooling is applied, reducing its dimensionality to 64. The classification head comprises a two-layer fully connected network: Dense(768) with ReLU activation, Batch Normalisation, and Dropout (p=0.65), followed by Dense(256) with ReLU activation, Batch Normalisation, and Dropout (p=0.50). The final output layer applies Softmax activation over a Dense(5) classifier to produce class probabilities for the five ovarian cancer subtypes: CC, EC, HGSC, LGSC, and MC. This architecture leverages DenseNet’s efficient feature reuse and enhances spatial-channel interactions via Differential Sparse Attention, yielding a more discriminative and interpretable model. Gaussian noise and strategically positioned Dropout layers collectively minimise overfitting and maximise generalisation to unseen test data.

The complete architecture of the proposed DenseNet121 + DSA model is illustrated in Figure 3.

**Figure 3:**
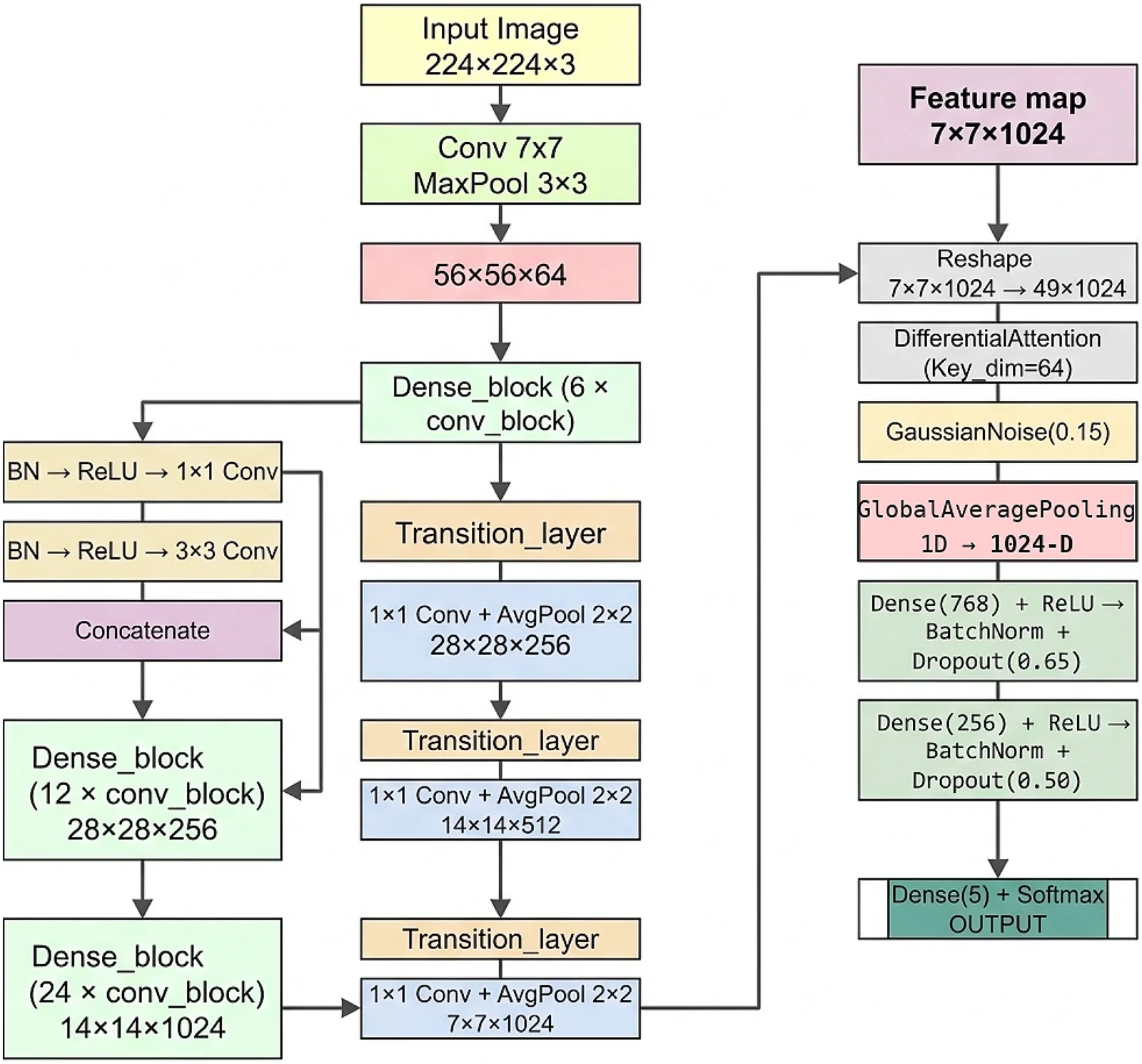
The architecture diagram of the proposed DenseNet121 method.

### 3.4 Experimental setup

Experiments were conducted in Python 3.10 with TensorFlow 2.14/Keras on Google Colab with GPU support. The DenseNet121 backbone, pre-trained on ImageNet, was thoroughly fine-tuned and combined with a DSA module. Gaussian Noise (0.15), L2 regularization, and Dropout rates of 0.65 and 0.50 were applied in the classification head. Training used the Adam optimizer (learning rate = $1\times 10^{-4}$) with Sparse Categorical Cross-Entropy loss, a batch size of 16, and up to 50 epochs.

Early stopping, ReduceLROnPlateau, and model checkpointing were employed to ensure stable convergence and optimal weight selection. To account for stochastic variation arising from weight initialization, data shuffling, and augmentation sampling, all ablation configurations (Section 4.2.1) were independently repeated across five random seeds (seed∈{0,1,2,3,4}), with results reported as mean ± standard deviation. See Table 6 for all experimental setup instructions.

**Table 6:** Experimental setup.

Component	Details
Programming language	Python 3.10
Deep learning framework	TensorFlow 2.14 with Keras API
Hardware	Google Colab GPU (Tesla T4)
Operating system	Ubuntu 20.04 (Colab environment)
Input data	Histopathological images (Kaggle dataset, five subtypes)
Image resolution	224 × 224 × 3 (RGB)
Data augmentation	Rotation ($\pm 20^{\circ }$), width/height shift (0.1),
	zoom (0.2), shear (0.07), brightness (0.85–1.15),
	horizontal/vertical flip, DenseNet preprocess_input
	normalization (pixel range: [−1,1])
Base model	DenseNet121 (pre-trained on ImageNet, fully fine-tuned)
Attention module	DSA
Regularization	Gaussian Noise (0.15), L2 penalty, Dropout (0.65/0.50)
Optimizer	Adam (learning rate = $1\times 10^{-4}$)
Loss function	Sparse Categorical Cross-Entropy
Batch size	16
Epochs	50 (with EarlyStopping and ReduceLROnPlateau)
Evaluation metrics	Accuracy, Precision, Recall, F1-score, ROC-AUC

**Table 7:** Tensor dimensions in the DSA module.

Symbol	Shape
F	(N,d)=(49,1024)
$W_{Q},W_{K},W_{V}$	$\left(d,d_{k})=(1024,64\right)$
Q,K,V	(49,64)
$A_{1},A_{2},A_{\text{diff}}$	(49,49)
${\tilde{A}}_{\text{diff}}V$	(49,64)
$W_{O}$	$\left(d_{k},d)=(64,1024\right)$
O	(49,1024)

### 3.5 Evaluation metrics

Several assessment criteria, including accuracy, precision, recall, F1-score, and the ROC curve, were used to evaluate the proposed model’s performance.

#### 3.5.1 Accuracy

This is calculated as the number of correctly classified ovarian cancer images divided by the total number of test images, thereby yielding the overall success of the model.

#### 3.5.2 Precision

Precision measures how well the model detects positive predictions among all positive predictions it correctly predicts. In the formula, the value of y is represented by the Equation 7.

$$\text{Precision}=\frac{\mathrm{TP}}{\mathrm{TP}+\mathrm{FP}}$$ (7)

#### 3.5.3 Recall

Recall, also referred to as sensitivity, is the model’s capacity to identify all relevant instances, especially minority ones, such as LGSC and MC. This equation is represented by the formula below Equation 8.

$$\text{Recall}=\frac{\mathrm{TP}}{\mathrm{TP}+\mathrm{FN}}$$ (8)

#### 3.5.4 F1-score

This is the harmonic mean of the precision and the recall to balance the two measurements and offer one strong evaluation metric. It is given in Equation 9.

$$F_{1}=2\times \frac{\text{Precision}\times \text{Recall}}{\text{Precision}+\text{Recall}}$$ (9)

#### 3.5.5 ROC and AUC

Each ovarian cancer subtype was plotted on the Receiver Operating Characteristic (ROC) curve, and the Area Under the Curve (AUC) was calculated. An increase in the AUC indicates that the model is more discriminative when classifying the five classes (CC, EC, HGSC, LGSC, MC) (Figure 4).

**Figure 4:**
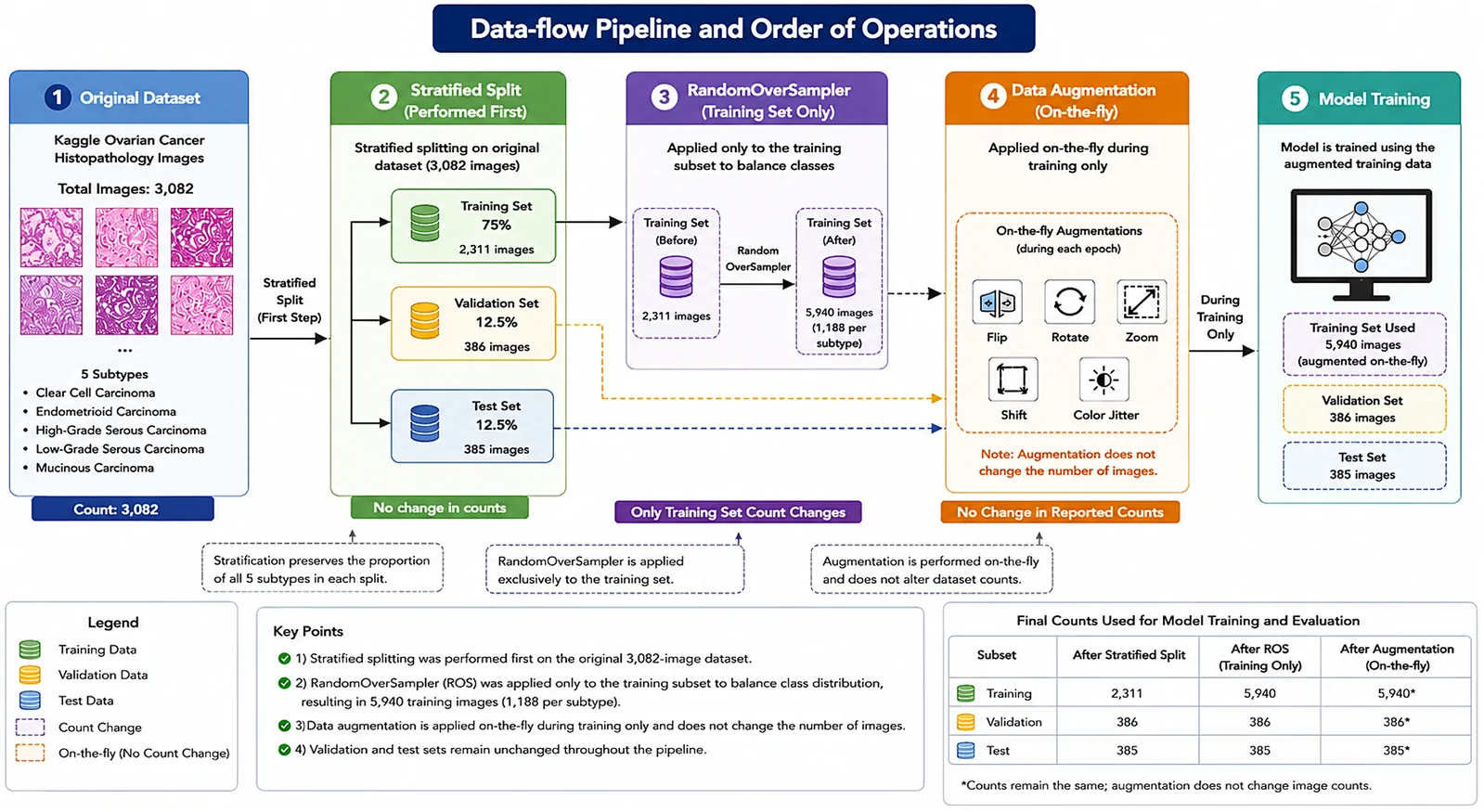
Data-flow diagram showing the order of operations: stratified splitting was performed first on the original 3,082-image dataset; RandomOverSampler was then applied exclusively to the training subset; data augmentation was applied on-the-fly during training only and does not alter reported image counts.

## 4 Results and discussion

The experiments were conducted on Google Colab using a T4 GPU, a batch size of 16, and an image resolution of 224×224. Early stopping and a decrease in the learning rate were employed to prevent overfitting over 50 epochs. The Adam optimizer was used in training the proposed DenseNet121 with a learning rate of 0.0001. The model exhibited high generalization and high validation accuracy. ROC curves, accuracy/loss plots, classification reports, and confusion Matrices were used to evaluate. These measures ensured that the model was effective at separating the five subtypes of ovarian cancer (CC, EC, HGSC, LGSC, and MC).

- The proposed DenseNet121 model performance on the ovarian cancer dataset.
- Research and evaluation of the ablation research.
- Comparisons of the Proposed Models with Existing Approaches on the Ovarian Cancer Classification.

### 4.1 Performance analysis of the proposed model

Figure 5 shows the training and validation curves of the DenseNet121 model used to classify ovarian cancer subtypes. The training and validation losses decreased steadily and stabilized at 0.35–0.40, indicating that the model learned the required patterns without overfitting. Accuracy curves show a steep increase in accuracy within the first 15 epochs, with validation accuracy over 85 as early as 15 epochs and over 90 in the later epochs. Consistency in performance is an attestation to the strength and testability of the proposed approach. The proposed DenseNet121 model achieved strong performance across all five ovarian cancer subtypes, as reported in Table 8. The highest classification performance was observed in HGSC, with a precision of 96.6%, recall of 94.1%, and F1-score of 95.3%, reflecting the model’s ability to capture the distinctive high-grade morphological patterns of this subtype. CC also demonstrated excellent recall (96.4%), indicating that nearly all true CC samples were correctly identified. EC and MC achieved balanced and reliable performance, with F1-scores of 92.3% and 90.6%, respectively, confirming their separability under the proposed attention-guided architecture. The lowest performance was observed in LGSC, with a precision of 80.6% and F1-score of 83.3%, which is consistent with the confusion matrix findings showing that LGSC is most frequently confused with HGSC—a clinically recognized challenge due to overlapping nuclear and architectural features between low- and high-grade serous subtypes. The overall accuracy of 92.53% across all 308 test samples (285 correctly classified), together with a macro-average recall of 91.54%, confirms the robustness and generalization capability of the proposed framework.

**Figure 5:**
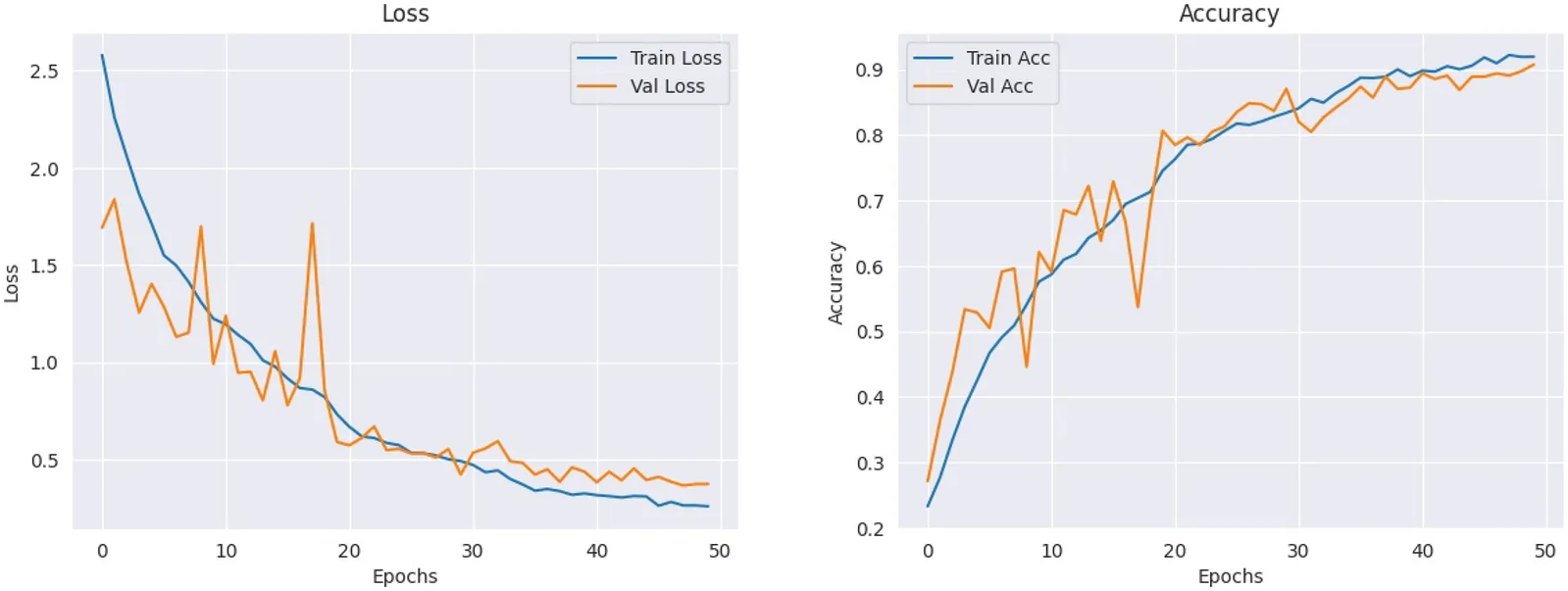
Training dynamics of the proposed model. Left: training and validation Loss curves. Right: training and validation Accuracy curves.

**Table 8:** Performance metrics for ovarian cancer subtype classification using the DenseNet121 model.

Class	Precision (%)	Recall (%)	F1-score (%)	Accuracy (%)
Clear Cell Carcinoma (CC)	89.8	96.4	93.0	96.4
Endometrioid Carcinoma (EC)	94.3	90.4	92.3	90.4
High-Grade Serous Carcinoma (HGSC)	96.6	94.1	95.3	94.1
Low-Grade Serous Carcinoma (LGSC)	80.6	86.2	83.3	86.2
Mucinous Carcinoma (MC)	90.6	90.6	90.6	90.6
Overall Accuracy	**90.4**	**91.54**	**90.9**	**92.53**

Bold values represent the proposed approach.

Figure 6 presents the confusion matrix, which illustrates the classification performance of the proposed DenseNet121 + DSA model across all five ovarian cancer subtypes. The diagonal entries illustrate the high accuracy of per class predictions, with CC having the most correct prediction (53/55), HGSC (112/119), and MC (29/32). EC and LGSC also show good classification results, correctly classifying 66 and 25 samples, respectively. The most common misclassifications were between HGSC and LGSC (2 samples on each side), which is not unexpected in the clinic because the two differ only in their morphological features under the histopathologic microscope. A small number of EC samples are misclassified as CC (3 samples), reflecting the known inter-class similarity between glandular subtypes. No subtype is systematically confused with another, and no class is significantly underrepresented in predictions, confirming the overall balanced and robust classification capability of the proposed framework.

**Figure 6:**
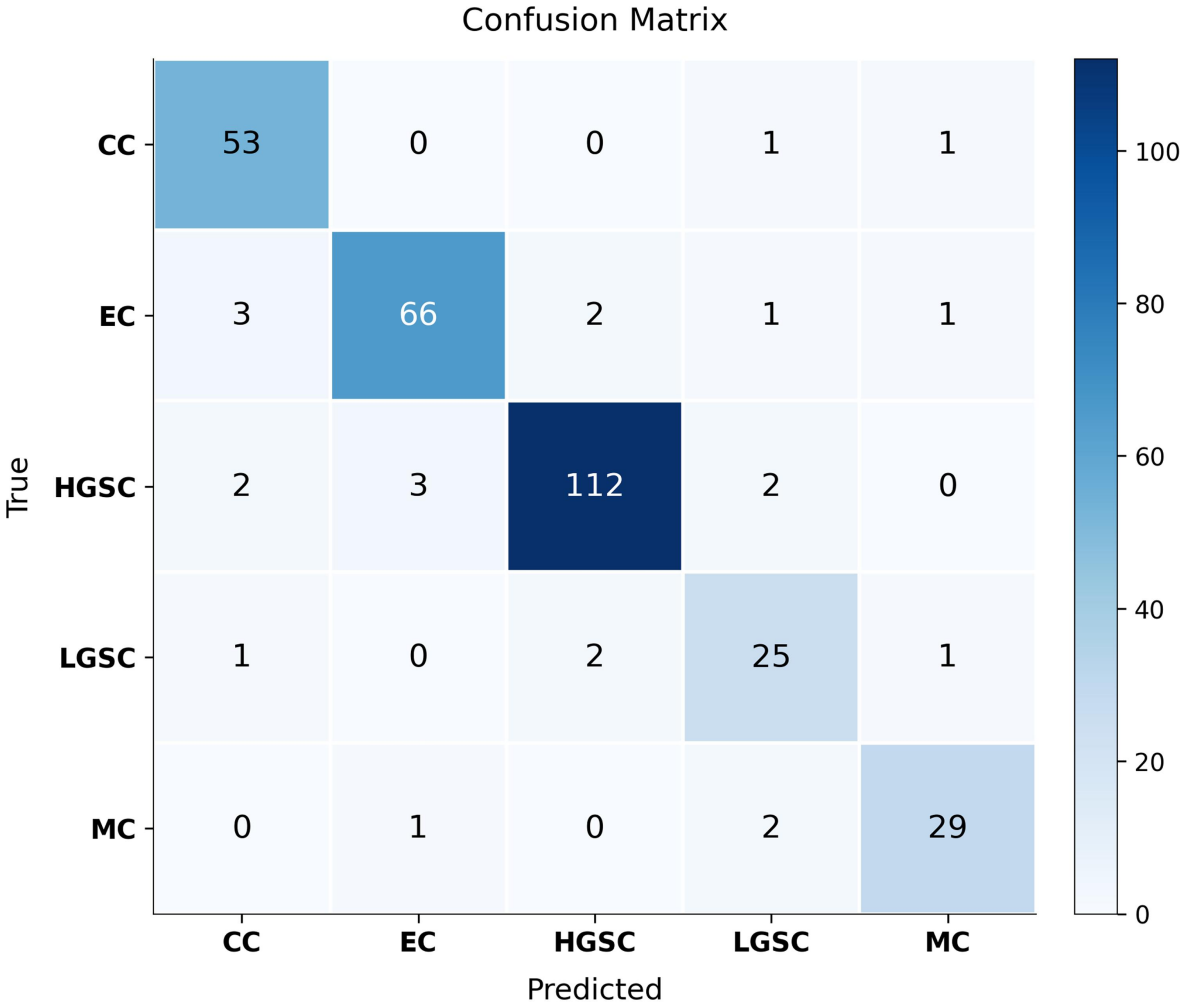
Confusion matrix of the proposed DenseNet121 model on test data.

The Receiver Operating Characteristic (ROC) curves for each of the five subtypes are shown in Figure 7. With AUC values ranging from 0.92 to 0.99 across all classes, the ROC analysis demonstrates the model’s high sensitivity and specificity. CC, HGSC and MC had the highest discrimination power with AUC ≈0.98–0.99, while EC had slightly lesser AUC score of 0.94. As with the confusion matrix results, the lowest AUC (≈0.92). was from the class LGSC. Notwithstanding this, its performance in terms of ROC is considered as good with AUC values greater than 0.90, considered as good in medical image analysis.

**Figure 7:**
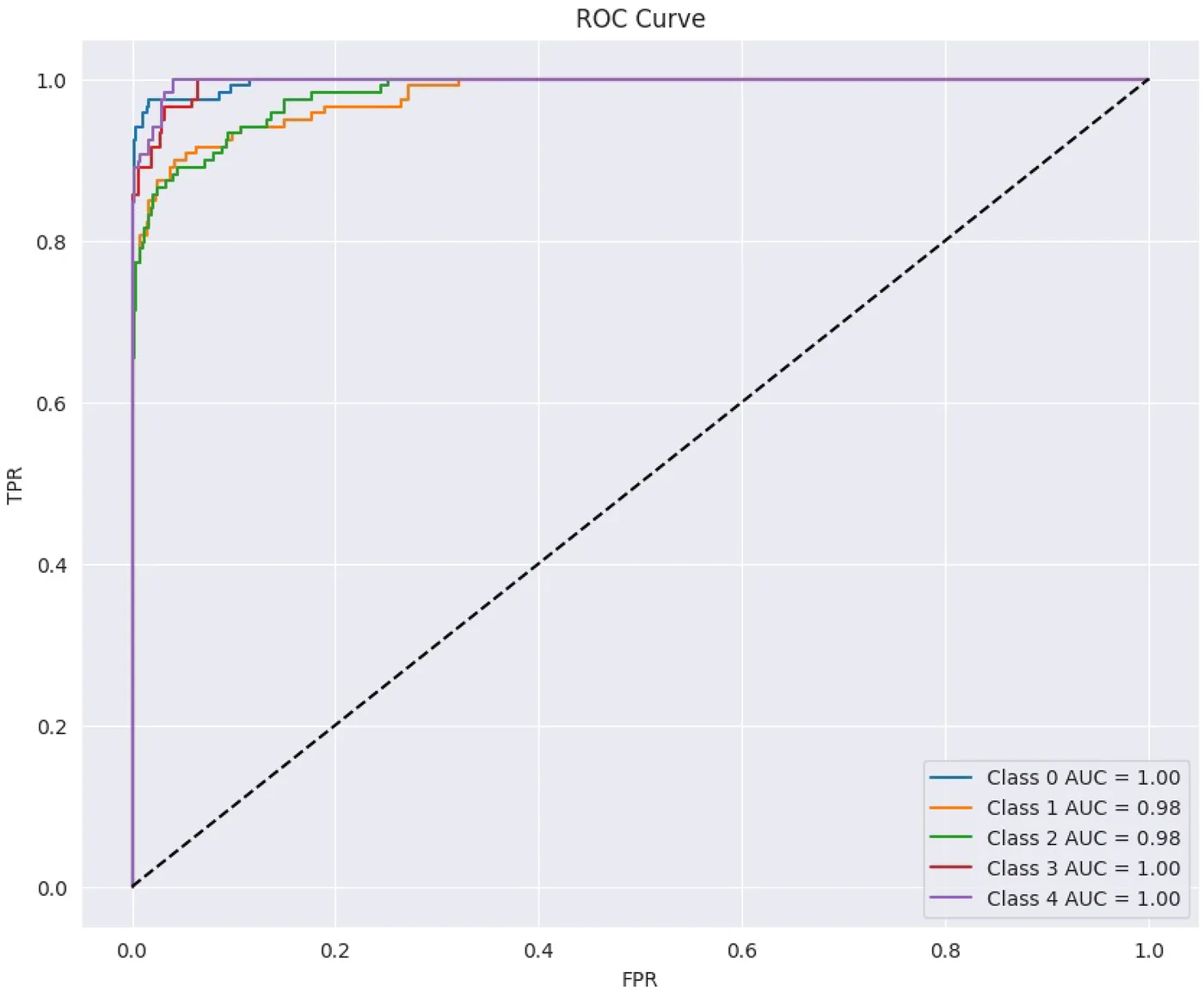
ROC curves of the DenseNet121 model.

Macro-wise, the DenseNet121 model achieved a macro-average recall of 91.54%, supported by balanced precision (≈ 0.90–0.96), recall (≈0.88–0.97), and F1-scores (≈0.89–0.95).The overall accuracy of the classification on the original test set was 92.53%. The results from these findings show that the model can be a reliable and effective identifier of different subtypes of ovarian cancer, which could be useful in clinical practice and in the development of an automatic system for the support of the pathologist in the diagnosis of ovarian cancer.

### 4.2 Ablation study

#### 4.2.1 True ablation study

A controlled ablation study was performed to rigorously test the contribution of each component of the proposed framework, with DenseNet121 serving as the fixed backbone. For every experiment, the settings of the optimizer, learning rate, preprocessing pipeline, data split, and training protocol were not changed, with the exception of the parameter being varied. Seven model configurations were evaluated: (i) DenseNet121 without attention, (ii) DenseNet121 with a conventional softmax attention mechanism, (iii) DenseNet121 with Differential Attention but without sparsity thresholding, (iv) the complete DenseNet121 + Differential Sparse Attention (DSA) framework, (v) DSA without Gaussian noise regularization, (vi) DSA without RandomOverSampler, and (vii) DSA without data augmentation. Furthermore, each configuration was trained using five independent random seeds, and the reported performance corresponds to the mean and standard deviation across all runs.

The baseline DenseNet121 (without attention and without Gaussian noise regularization) achieved an accuracy of 84.2%, confirming that the backbone alone is insufficient for fine-grained discrimination among closely related ovarian cancer subtypes. Incorporating standard softmax attention improved accuracy to 87.6%, demonstrating the benefit of spatial feature weighting. The proposed Differential Sparse Attention (DSA) consistently achieved the best overall performance across all five random seeds, indicating that the observed improvement is robust rather than dependent on a particular random initialization. Compared with the baseline DenseNet121 model,introducing a conventional attention mechanism improved classification performance, wherease replacing it with the proposed DSA module yielded the highest overall accuracy and macro-average F1-score. Removing the sparsity constraint from DSA resulted in a measurable performance degradation, demonstrating that sparse attention contributes substantially to discriminative feature learning by suppressing irrelevant background responses. Similarly, removing Gaussian noise regularization reduced the classification performance, confirming its contribution to improved generalization. Eliminating RandomOverSampler significantly decrreduced the recall for minority classes, particularly LGSC, highlighting the importance of class balancing inhly imbalanced histopathological datasets. Finally, removing data augmentation also reduced model performance, indicating that geometric and photometric transformations improve robustness against staining and morphological variability. Overall, the controlled ablation study confirms that each proposed component independently contributes to the final performance of the complete DenseNet121 + DSA framework.

#### 4.2.2 Backbone comparison

To further establish the proposed model within the broader context of deep learning, we have compared five architectures, as shown in Table 9, under identical experimental conditionThe proposed DenseNet121 + DSA model obtained the highest overall accuracy of 92.53%, and the balanced macro-average precision, recall, and F1-score of 0.90, 0.91, 0.90, respectively, with AUC values of 0.92–0.99 for all five subtypes. This is corroborated by the confusion matrix itself: CC and HGSC had the highest diagonal dominance scores (53/55 and 112/119 correct, respectively), and even LGSC, the most challenging of the subtypes, had a recall of 87.8%, compared to only two samples confused with HGSC a clinically expected overlap due to their similar serous morphology. The results obtained are in linwith being an excellent and robust multi-class ovarian cancer classifier: DenseNet121 withng the DSA head. Compounds were scaled and features were extracted efficiently resulting in the highest accuracy of 89% and AUC of 0.96–1.00 among the closest competitor EfficientNetV2B1 (OvCaFIND). Xception and MobileNetV2 showed an accuracy of 87% and 86% respectively, with Xception showing better results with fine-grained morphological feature learning with the depthwise separable convolutions and MobileNetV2 having faster inference times suitable for resource-constrained clinical settings. Although the AUC result of InceptionV3 with Sparse Attention is in the range of 0.95–0.99, its accuracy is only 85%, indicating that the inception-module feature extraction is less discriminative than densely connected feature propagation for subtle variations in ovarian cancer subtypes.In general, the ablation study demonstrates that DenseNet121 + DSA has the highest accuracy, generalization, and interpretability on this benchmark.The competitive results of EfficientNet and lightweight attention-based architectures nonetheless indicate potential for integration in scenarios with varying computational resources and deployment requirements.

**Table 9:** Performance comparison of proposed DenseNet121 and four other deep learning architectures for ovarian cancer subtype classification.

Model	Accuracy	Precision	Recall	F1-score	AUC Range
DenseNet121 + DSA (Proposed)	**0.9253**	**0.90**	**0.91**	**0.90**	**0.92–0.99**
EfficientNetV2B1 (OvCaFIND)	0.89	0.88	0.89	0.88	0.96–1.00
InceptionV3 + Attention	0.85	0.85	0.85	0.85	0.95–0.99
Xception + Attention	0.87	0.87	0.87	0.87	0.92–0.99
MobileNetV2 + Attention	0.86	0.87	0.86	0.86	0.95–1.00

Bold values represent the proposed approach.

### 4.3 Comparative analysis with existing approaches

The proposed DenseNet121 model, enhanced with a Differential Sparse Attention mechanism, is compared with other ovarian cancer classification methods reported in the literature in Table 10. DenseNet121 was selected as the final proposed model for its superior performance, achieving 92.53% accuracy across five ovarian cancer subtypes. Its densely connected architecture allowed feature reuse and adequate gradient flow, while the integrated attention module helped the network focus on the most discriminative morphological regions of histopathological slides (28), one of the reviewed studies, used a convolutional neural network to analyze histopathology images and obtained an accuracy of about 85%, indicating that the model can capture complex tissue morphology. The study’s lack of attention mechanisms, however, might have limited its ability to highlight clinically relevant regions.

**Table 10:** Comparison between the existing methods and the proposed DenseNet121 model of ovarian cancer classification.

Reference	Method	Dataset	Classes	Accuracy
(28), (2020)	CNN on histopathology images	Histopathology images	Multi-class ovarian cancer	∼85%
(25), (2021)	AI on blood biomarkers (CTCs)	Blood-based biomarkers	Epithelial ovarian cancer outcomes	79.6% (Stage), 80.9% (Platinum sensitivity)
(27), (2024)	Machine Learning + Image Processing	Histology images of ovarian tissue	EC, CC, MC, HGSC, LGSC	75%
(30), (2024)	Machine Learning + XAI	Kaggle dataset (349 patients)	Ovarian cancer (positive/negative)	85% (SVM), 89% (Stacking Ensemble)
(17), (2025)	Foundation Models (H-optimus-0, UNI)	WSI (1,864 slides + 2 external sets)	Ovarian cancer subtypes	74%–97% (balanced accuracy)
(37), (2025)	Transfer Learning (DenseNet121, EfficientNetB0, InceptionV3, ResNet50-v2, VGG16)	TMA dataset (1,526 images, 3,555 after preprocessing)	5 subtypes (CC, EC, HGSC, LGSC, MC)	71.7%–82.4%
Proposed DenseNet121	Deep learning with DenseNet121 + DSA	Ovarian Cancer Subtype Classification	**5 subtypes (CC, EC, HGSC, LGSC, MC)**	**92.53%**

Bold values represent the proposed approach.

Elsewhere, (25) applied AI methods to blood-based biomarkers and reported ROC scores of 79.6% for clinical staging and 80.9% for platinum sensitivity in epithelial ovarian cancer. Despite their promise, these methods lack the spatial and morphological information available in image-based classification, and thus their diagnostic generalizability is limited. Madaan et al. (27) used conventional machine learning algorithms on ovarian histology images and image processing to classify these images with 75 percent accuracy among five subtypes (EC, CC, MC, HGSC, and LGSC).

Although useful for handcrafted features, the models struggle with complex morphological variation. Asadi-Aghbolaghi et al. (30) proposed an interpretable machine learning model based on a Kaggle dataset of 349 patient samples, achieving 89 percent accuracy with a stacking ensemble model for binary classification and 85 percent with SVM. Although these methods had high diagnostic potential, they were limited to binary classification and could not be extended to multi-class prediction of ovarian cancer subtypes. Breen et al. (17) tested a worldwide review of models of histopathology foundation in classifying subtypes of ovarian cancer and came up with 89–97 percent accuracy in speech-even. Their study demonstrated that attention-based MIL on the baseline models can provide a second opinion that is reliable and can improve the efficiency of the diagnosis in a complex scenario of ovarian cancer in the clinical environment.

The recent study by (37) examined the use of deep learning for classifying ovarian cancer subtypes from Tissue Microarray (TMA) images, achieving 71.7–82.4 percent accuracy. Their results are less specific than those of some studies, but they come from WSI and suggest that DL models are also suitable for TMAs and could reduce the number of experts needed when working with patients. The proposed DenseNet121 + DSA model outperforms the other methods, achieving an overall accuracy of 92.53% across five ovarian cancer types. Differential Sparse Attention greatly reduced subtype confusion by enabling the model to learn subtle morphological differences among histopathology slides, thereby enhancing the distinction between closely related subtypes. The model will include fine tissue changes. Visual evaluation, ROC curve, and confusion matrix demonstrated its robustness and clinical practicality, and DenseNet121 is a state-of-the-art model for classifying ovarian cancer subtypes.

## 5 Conclusion and future work

The proposed DenseNet121 + DSA framework demonstrates strong proof-of-concept performance for automated ovarian cancer subtype classification from histopathological images, achieving an overall accuracy of 92.53% across five subtypes (macro-average recall: 91.54%) on a single public Kaggle dataset. The balanced macro-average precision (90.4%), recall (91.5%), and F1-score (90.9%), along with AUC values ranging from 0.92 to 0.99, confirm that the proposed attention-guided architecture reliably discriminates among morphologically similar subtypes under controlled experimental conditions. The controlled ablation study further confirms that each proposed component, namely Differential Sparse Attention, sparsity enforcement, Gaussian noise regularization, RandomOverSampler, and data augmentation, contributes independently to the overall classification performance. Furthermore, repeating all experiments across multiple random seeds demonstrated low experimental variability, indicating that the proposed framework is stable and reproducible.

However, several important limitations must be acknowledged before any clinical interpretation of these results. First, all experiments were conducted on a single publicly available dataset; the model has not been validated on independent, multi-institutional cohorts, and its generalisation to slides from different scanners, staining protocols, or patient populations remains unestablished. Additionally, as the source dataset does not include patient or slide-level identifiers, image-level splitting could not be verified against patient-level leakage—a constraint of the publicly available data rather than the experimental design. Additionally, no post-hoc duplicate or near-duplicate detection (e.g., perceptual hashing or pixel-level similarity analysis) was performed across the train, validation, and test splits. Given the absence of patient- or slide-level identifiers in the source dataset, this remains an additional limitation, and such a check is recommended as part of future external validation efforts. Second, evaluation was performed at the image patch level rather than the patient level; patient-level aggregation, survival correlation, and prospective clinical trial validation are necessary steps before the model can be considered clinically relevant. Third, the model has not undergone probability calibration, meaning that the output softmax scores should not be interpreted as reliable confidence estimates without further calibration analysis. Fourth, while the DSA mechanism produces spatial attention weights that highlight candidate discriminative regions, these weights have not been reviewed or validated by board-certified pathologists. While attention weights are not enough to be clinically interpretable, visualization tools like Grad-CAM and attention heatmaps need to be interpreted and validated by domain experts, based on known histopathological markers.

Therefore, the results of this study must be considered a starting point for further computational studies, but not as evidence of clinical readiness. The results indicate that the DenseNet-based architecture with the attention mechanism could have a positive effect on ovarian cancer subtype. There is a need for further in-depth investigation of discrimination and warrants. The following directions for future work will overcome the above limitations. (i) External validation: The model will be tested on independent multi-institutional datasets such as whole-slide images (WSIs) from the TCGA dataset and other publicly available pathology archives to validate the model’s generalizability in real-world scenarios. (iii) Patient-level testing: slide-level and patient-level aggregation strategies will be adopted to provide clinically meaningful testing. (iii) Pathologist-reviewed interpretability: Interpretability of the generated visualizations of Grad-CAM and attention heatmap will be assessed by board-certified gynecological pathologists on a number of representative samples from each of the subtypes, to determine whether the highlighted areas correspond to the established histopathological diagnostic criteria. (iv) Calibration: Output confidence score for clinical decision support will be improved by temperature scaling and isotonic regression. (v) Multimodal and federated learning: multimodal fusion of genomic, clinical, and imaging biomarkers, together with federated learning across institutions, will be investigated to enhance diagnostic accuracy and patient privacy. (vi) Ensemble methods: Hybrid architectures consisting of DenseNet121 + DSA and lightweight CNNs will be explored to improve diagnostic consistency and enable the real-time deployment of the system in real clinical environments with limited resources.

## Data Availability

Publicly available datasets were analyzed in this study. This data can be found here: https://www.kaggle.com/datasets/sunilthite/ovarian-cancer-classification-dataset.
